# HIV-1 matrix protein p17 and its variants promote human triple negative breast cancer cell aggressiveness

**DOI:** 10.1186/s13027-017-0160-7

**Published:** 2017-09-25

**Authors:** Francesca Caccuri, Francesca Giordano, Ines Barone, Pietro Mazzuca, Cinzia Giagulli, Sebastiano Andò, Arnaldo Caruso, Stefania Marsico

**Affiliations:** 1 Section of Microbiology, Department of Molecular and Translational Medicine, University of Brescia , Brescia, Italy; 2 Department of Pharmacy, Health and Nutritional Sciences, University of Calabria, Arcavacata di Rende, Italy

**Keywords:** HIV-1 matrix protein p17, p17 variants, Breast cancer, Motility, Clonogenicity, ERK1/2 signaling pathway

## Abstract

**Background:**

The introduction of cART has changed the morbidity and mortality patterns affecting HIV-infected (HIV^+^) individuals. The risk of breast cancer in HIV^+^ patients has now approached the general population risk. However, breast cancer has a more aggressive clinical course and poorer outcome in HIV^+^ patients than in general population, without correlation with the CD4 or virus particles count. These findings suggest a likely influence of HIV-1 proteins on breast cancer aggressiveness and progression. The HIV-1 matrix protein (p17) is expressed in different tissues and organs of successfully cART-treated patients and promotes migration of different cells. Variants of p17 (vp17s), characterized by mutations and amino acid insertions, differently from the prototype p17 (refp17), also promote B-cell proliferation and transformation.

**Methods:**

Wound-healing assay, matrigel-based invasion assay, and anchorage-independent proliferation assay were employed to compare the biological activity exerted by refp17 and three different vp17s on the triple-negative human breast cancer cell line MDA-MB 231. Intracellular signaling was investigated by western blot analysis.

**Results:**

Motility and invasiveness increased in cells treated with both refp17 and vp17s compared to untreated cells. The effects of the viral proteins were mediated by binding to the chemokine receptor CXCR2 and activation of the ERK1/2 signaling pathway. However, vp17s promoted MDA-MB 231 cell growth and proliferation in contrast to refp17-treated or not treated cells.

**Conclusions:**

In the context of the emerging role of the microenvironment in promoting and supporting cancer cell growth and metastatic spreading, here we provide the first evidence that exogenous p17 may play a crucial role in sustaining breast cancer cell migration and invasiveness, whereas some p17 variants may also be involved in cancer cell growth and proliferation.

## Background

In the era of cART, a change has emerged in the type of cancers affecting HIV-1-infected (HIV^+^) patients [1]. The incidence of the three classically AIDS-related cancers (Kaposi’s sarcoma, non-Hodgkin lymphoma and cancer of the cervix) is greatly decreased [2], whereas recent clinical studies in the HIV^+^ population have shown a significant increase of some Non-AIDS Defining Cancers (NADCs) [3, 4]. including breast cancer [5, 6].

Breast cancer is the most frequently diagnosed malignancy in women and the leading cause of cancer death among females in economically developing countries [7]. In early time, the risk of breast cancer in the population with AIDS had been lower than in the general population [8, 9], with small variations in incidence relative to the CD4 count or duration of infection [10]. Today, breast cancer risk in women with AIDS has been increasing and approaching to general population risk [11–14]. More interesting, recent studies have evidenced a more aggressive clinical course, poorer outcome and younger age at diagnosis of breast cancer in the HIV-1 setting compared to general population [15–17]. These findings suggest a likely influence of HIV-1 on breast cancer progression, although the retrovirus does not show any capability to exert a direct tumorigenic effect on this cancer [15].

Numerous studies have shown a direct involvement of viral proteins in carcinogenesis [18–21]. Many viruses encode cytokine homologues that bind to host specific receptors triggering signal transduction cascades and biological responses including activation and proliferation of target cells, thus contributing directly to the cancer associated with viral infection [22]. The HIV-1 matrix protein p17 (p17) is a structural protein with a well-established role in the virus life cycle [23]. It is easily detected in the plasma and tissue specimens of HIV^+^ patients [24, 25] even in patients under successful cART and in the absence of any in situ viral replication [26, 27]. This is not surprising since latently HIV-1-infected resting T cells are capable of producing HIV-1 proteins without supporting spreading of infection [28]. Moreover, recent data show the capability of *Gag*-expressing cells to release p17 in the absence of viral protease, following its cellular aspartyl proteases-dependent cleavage from the *Gag* precursor protein [29]. Overall, these findings indicate that p17 can be produced by cells potentially residing in different tissues and organs, even in the absence of viral replication. Extracellularly, p17 has been found to deregulate the biological activity of different cells that are directly or indirectly involved in AIDS pathogenesis [30–35]. Moreover, we have provided evidence that a p17 variant derived from a Ugandan HIV-1 strain A1 (S75X) triggers an activation of the PI3K/Akt signaling pathway in B-cells, compared to a prototype p17 isolated from clone BH10 of clade B (refp17). As a consequence, the p17 variant S75X was found to increase B-cell proliferation and clonogenicity, providing the first evidence on the existence of p17 natural variants with B-cell transforming activity [36]. More recently, p17 variants (vp17s) endowed with B-cell clonogenic activity, and characterized by amino acid insertions at the C-terminal region of the viral protein, were more frequently detected in plasma of HIV^+^ patients with than without non-Hodgkin lymphomas (HIV-NHL) [37], focusing our attention on their potential role in lymphomagenesis.

Exogenous p17 binds to CXCR1 and CXCR2 [25, 30], two seven-transmembrane G-protein-coupled receptors for IL-8. Consequently, p17 mimics IL-8 activity on cells expressing these receptors on their surface. Breast cancer cells do express CXCR1 and CXCR2 [38], and increasing evidence indicates that IL-8 plays a critical role in enhancing the invasive and metastatic potential of breast cancer cells [39, 40]. Moreover, targeting IL-8 receptors has proven efficacious in in vivo models of breast cancer, as well as in primary invasive and metastatic breast cancer [41].

All these findings suggest a possible association between p17 and/or its variants expression in tissue microenvironment and breast cancer aggressiveness in HIV^+^ individuals. The aim of present study was to investigate the biological activity of p17 and its variants on the triple-negative (ER^−^, PR^−^ and HER-2^−^) MDA-MB 231 cells as a model of human breast cancer.

## Methods

### Cell line and recombinant proteins

The human breast cancer cell line, MDA-MB 231, was obtained from the American Type Culture Collection (ATCC, Manassas, VA, USA) and grown as described. Purified endotoxin (lipopolysaccharide)-free recombinant refp17 (from clone BH10 of clade B isolate) and vp17s (namely NHL-a101, NHL-a102 and NHL-a105 derived from HIV^+^ patients with NHL) were produced as previously described [31, 37]. The absence of endotoxin contamination (< 0.25 endotoxin U/ml) in the proteins preparation was assessed by Limulus amoebocyte assay (Associates of Cape Cod Inc., East Falmouth, MA, USA).

### Wound healing assay

MDA-MB 231 cells were plated into 24-well plates (10^5^ cells/well) in complete medium. Confluent monolayers were nutrient starved by growing them for 24 h in medium containing 0.5% FBS and then scratched using a 200 μl pipette tip. After washing, cells were treated or not with 10 ng/ml of refp17 or vp17s in complete medium (10% FBS). When reported, starved MDA-MB 231 cells were pretreated with 2.5 μg/ml of mAb to CXCR1 (mAb 330; R&D, Minneapolis, MN, USA) or to CXCR2 (mAb 331; R&D), or with an isotype-matched mAb (2.5 μg/ml; R&D) for 1 h at 37 °C before proteins stimulation. In some experiments, MDA-MB 231 cells were serum starved for 24 h in the presence or absence of inhibitors of PI3K/Akt (LY294002) (20 μM) (ENZO Life Sciences, Farmingdale, NY, USA), Jak/STAT (AG-490) (20 μM) (Sigma-Aldrich, St. Louis, MO, USA) or MEK/ERK1/2 (PD98059) (10 μM) (Calbiochem, Billerica, MA, USA) signaling pathways. Cell migration was evaluated at different time points using an inverted microscope (DM-IRB microscope system, Leica, Buffalo Grove, IL, USA). Cells were photographed using a CCD camera (Hitachi Inc., Krefeld, Germany). Wound closure was monitored over 12 h. In some experiments, in order to count the cells migrating into the wound area or protruding from the border of the wound, cells were fixed before wound closure and stained with Comassie brilliant blue.

### Invasion assay

Cell invasion assay was carried out by the Matrigel-coated transwell system as previously described [42]. Polycarbonate transwell filters (8 μm pore size, Corning, Tewksbury, MA, USA) were coated with 50 μg of Cultrex® basement membrane extract (BME; 10 mg/ml; Trevigen, Gaithersburg, MD, USA) diluted in a total volume of 150 μl of serum-free medium. Then the transwells were placed in a 24 well/plate. Cells were seeded into the coated filter at a concentration of 10^4^ cells/well. Six hundred μl of medium containing or not 200 ng/ml of refp17 or vp17s were added into the lower chamber. The plate was incubated at 37 °C and after 48 h of incubation, cells that had crossed the filter were fixed, stained with Coomassie brilliant blue and counted. The percentage of invasion was calculated as follow: number of cells invading through Matrigel coated membrane/total number of seeded cells × 100.

### Western blot analysis

MDA-MB 231 cells were nutrient starved for 24 h and treated for 30 min with refp17 or vp17s at concentrations ranging from 50 to 200 ng/ml. When indicated, MDA-MB 231 cells were nutrient starved for 24 h in the presence or absence of PD98059 (10 μM) to inhibit the MEK/ERK1/2 signaling pathway. Cells were then lysed in 200 μl of lysis buffer [50 mM HEPES (pH 7.5), 150 mM NaCl, 1.5 mM MgCl_2_, 1 mM EGTA, 10% glycerol, 1% Triton X-100, protease inhibitors (Sigma-Aldrich)]. Equal amounts of total proteins were resolved on a 11% SDS-polyacrylamide gel and then blotted onto a nitrocellulose membrane. The blots were incubated overnight at 4 °C with rabbit polyclonal antibodies to pAkt (Ser473), Akt (Cell Signaling, Danvers, MA, USA), pSTAT3 (Tyr705), STAT3 (Cell Signaling), ERK1/2 (Santa Cruz Biotechnology, Inc., Santa Cruz, CA) or a mouse monoclonal antibody to pERK (Thr202, Tyr204) (Santa Cruz Biotechnology, Inc.). The antigen-antibody complex was detected by incubation of the membranes for 1 h at room temperature with peroxidase-conjugated goat anti-rabbit IgG or goat anti-mouse IgG (Thermo Scientific, Waltham, MA, USA) and revealed using the Enhanced Chemiluminescence System (ECL System, Santa Cruz Biotechnology, Inc.).

### MTT assays

Cell viability was evaluated with the MTT assay (Sigma-Aldrich). MDA-MB 231 cells were seeded in 24-well plates at a density of 2 × 10^4^/well and then treated with refp17 or vp17s, at the indicated concentrations, in phenol red-free medium containing 5% cs-FBS. Forty-eight h after the beginning of proteins stimulation, 100 μl of the MTT stock solution (2 mg/ml) were added to each well and the plate was incubated for 2 h at 37 °C. The medium was then removed and cell lysis was carried out by adding 500 μl of DMSO (Sigma-Aldrich) and shaking the plates for 15 min on an orbital shaker. The absorbance was measured at 570 nm using the Beckman Coulter Spectrophotometer (Brea, CA, USA).

### Soft agar anchorage-independent growth assay

MDA-MB 231 cells (3 × 10^4^/well) were plated in 12-well plates in 2 ml of phenol red-free medium containing 0.35% Sea-Plaque agarose (Lonza, Amboise, France) and 5% cs-FBS, over a 0.7% agarose base. One day after plating, medium containing or not viral proteins was added to the top of the layer and replaced every 4 days. After 15 days, 300 μl of MTT (Sigma-Aldrich) were added to each well and allowed to incubate for 4 h at 37 °C. Plates were then placed overnight at 4 °C, and colonies >50 μm diameter were counted.

### Statistics

Data obtained from multiple independent experiments are expressed as the mean ± standard deviations (SDs). The data were analyzed for statistical significance by one-way or two-way ANOVA, when appropriate. Bonferroni’s post-test was used to compare data. Differences were considered significant at a *P* value of <0.05. Statistical tests were performed using Prism 5 software (GraphPad).

## Results

### Refp17 and its variants increase MDA-MB 231 cell migration

**Fig. 1:**
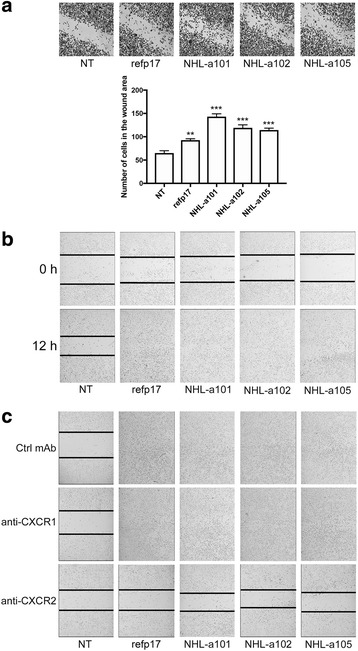
Refp17 and vp17s promote breast cancer cells migration. In the wound-healing assay, confluent MDA-MB 231 cell monolayers were serum starved for 24 h and then scratched using a 200 μl pipette tip. Cells were cultured in complete medium either unsupplemented or containing 10 ng/ml of refp17 or vp17s. **a** After 6 h of culture cells were fixed and stained with Coomassie brilliant blue. The cells migrated into the wound area were counted. **b** The percentage of wound healing was observed over a period of 12 h. **c** Wound healing assay was performed pretreating MDA-MB 231 cells for 1 h with a neutralizing mAb to CXCR1 (2.5 μg/ml), CXCR2 (2.5 μg/ml), or with an isotype-matched mAb (Ctrl mAb; 2.5 μg/ml). Images are representative of three independent experiments with similar results (original magnification 10×). Statistical analysis was performed by one-way ANOVA and the Bonferroni’s post-test was used to compare data (** *p* < 0.01, *** *p* < 0.001)

The ability of refp17 and vp17s to promote the migratory activity of MDA-MB 231 cells was assessed by wound healing assay. This method allows us to investigate the ability of viral proteins to modulate cell migration by sealing a confluent cell monolayer after mechanical injury. MDA-MB 231 cells were grown into 24-well plates and starved for 24 h. Confluent monolayers were scratched with a pipette tip and the percentage of wound healing was observed over a period of 12 h. The number of MDA-MB 231 cells in the wound area increased more quickly (Fig. 1a) and the wound area decreased more rapidly (Fig. 1b) in refp17- and vp17s-treated cells as compared to control cells. As shown in Fig. 1b, not treated (NT) MDA-MB 231 cells reached approximately 53% healing (range from 48 to 58%) after 12 h of culture, whereas at the same time cells treated with refp17 or with its variants NHL-a101, NHL-a102 or NHL-a105 reached 100% healing, showing a strong improvement in wound repair ability. To clarify the involvement of the known p17 receptors CXCR1 and CXCR2 in viral proteins-induced MDA-MB 231 cell motility, monolayers were pretreated for 1 h with 2.5 μg/ml of neutralizing mAb to CXCR1, CXCR2, or with 2.5 μg/ml of an isotype-matched mAb (Ctrl mAb). Immediately after pretreatment, confluent monolayers were scratched with a pipette tip and cultured for 12 h with or without 10 ng/ml of refp17, NHL-a101, NHL-a102 or NHL-a105. As shown in Fig. 1c, MDA-MB 231 cells pretreated with the isotype-matched mAb and then treated with viral proteins reached 100% of wound healing after 12 h of culture. Similar results were obtained when cells were pretreated with the neutralizing mAb to CXCR1, whereas pretreatment of cells with the neutralizing mAb to CXCR2 strongly inhibited cell migration promoted by all viral proteins. This finding suggests that refp17 and vp17s utilize CXCR2 to trigger breast cancer cell motility.

### Both refp17 and vp17s promote breast cancer cell invasion

**Fig. 2:**
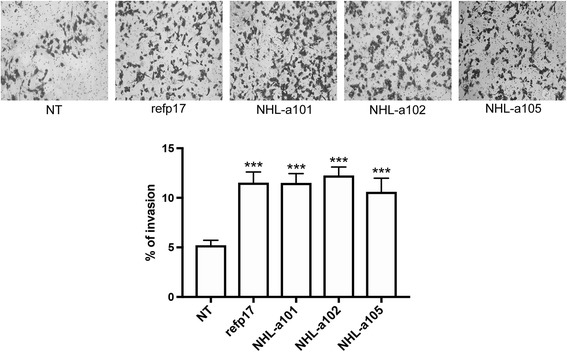
Refp17 and vp17s increase breast cancer cell invasion. Cell invasion assay was performed by matrigel-coated transwell system. Cells were resuspended in a serum-free medium and seeded in the upper chamber. Medium supplemented with 200 ng/ml of refp17 or vp17s was used as chemoattractant factor in the lower chamber. After 48 h of culture, migrated cells were stained, photographed and counted (original magnification 10×). Data represent the average of three independent experiments performed in triplicate. Images are representative of three independent experiments with similar results. Statistical analysis was performed by one-way ANOVA, and the Bonferroni post-test was used to compare data (*** *P* < 0.001)

The metastatic potential of tumor cells is largely dependent on their ability to degrade and migrate through the extracellular matrix. Therefore we also evaluated the effect of refp17 and vp17s on the migratory capacity of breast cancer cells using the Matrigel-based invasion assay. As shown in Fig. 2 (upper panel), invasion of MDA-MB 231 cells strongly increased upon viral proteins stimulation as compared to NT cells. Quantitative analysis showed that only 5.2% of NT cells were able to invade the matrigel and the filter compared to 11.7, 11.5, 12.2 and 10.6% of cells stimulated with refp17, NHL-a101, NHL-a102 and NHL-a105, respectively (Fig. 2, lower panel). This result suggests that MDA-MB 231 cells treated with refp17 and vp17s have a much higher invasive potential than control cells.

### Both refp17 and vp17s promote breast cancer cell migration through modulation of the ERK1/2 signaling pathway

**Fig. 3:**
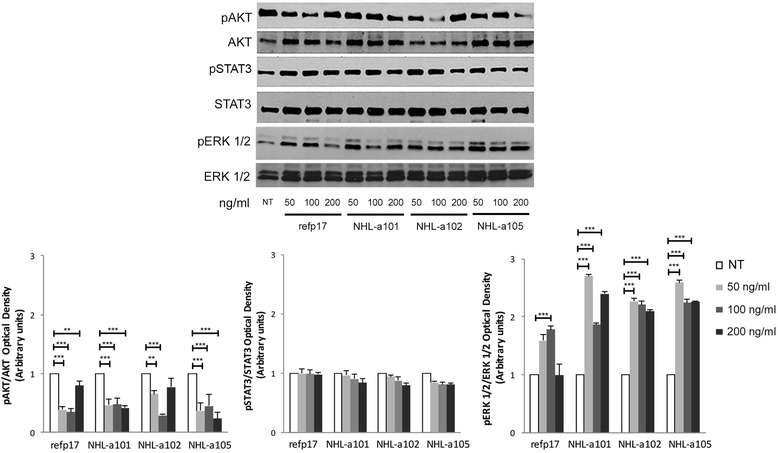
Effects of refp17 and vp17s on Akt, STAT3 and ERK1/2 activity in MDA-MB 231 cells. Cells were treated or not (NT) for 30 min with 50, 100 and 200 ng/ml of refp17 or vp17s and then lysed. Equal amounts of total cellular extracts were analyzed for expression of pAkt, Akt, pSTAT3, STAT3, pERK1/2 or ERK1/2 by western blot analysis using mAbs to pAkt (Ser473), Akt, pSTAT3 (Tyr705), STAT3, pERK1/2 (Thr202, Tyr204) or ERK1/2 as specific reagents. Phosphorylation of Akt, STAT3 and ERK1/2 was verified by densiometric analysis and plotting of the pAkt/Akt, pSTAT3/STAT3 and pERK1/2/ERK1/2. Upper panel, Blots from one representative experiment of three with similar results are shown. Lower panel, Values reported for Akt, STAT3 and ERK1/2 are the mean ± SD of three independent experiments. Statistical analysis was performed by one-way ANOVA, and the Bonferroni post-test was used to compare data (** *p* < 0.01, *** *p* < 0.001)

Western blot analyses were performed to determine whether refp17 and vp17s effects on MDA-MB 231 cells were mediated by modulation of signaling pathways usually involving tumor cell motility and invasion. We investigate the ability of refp17 and its variants to modify the phosphorylation status of Akt, STAT3 and ERK1/2 of MDA-MB 231 cells. As shown in Fig. 3, breast cancer cells stimulated with refp17 or vp17s showed a significant activation of ERK1/2 compared to NT cells, as evidenced by up-regulation of phosphorylated ERK1/2. On the other hand, all viral proteins did not exert any effect on the phosphorylation status of STAT3, indicating that this pathway is not involved in refp17 and vp17s activity on MDA-MB 231 cells. Surprisingly, inhibition of Akt activation occurred upon stimulation of tumor cells with both refp17 and vp17s. Altogether, our findings are consistent with a potential link between increase of migration and invasion of refp17 and its variants and modulation of ERK1/2 and Akt pathways.

### Refp17 and vp17s breast cancer cells promoting activity is specifically mediated by ERK1/2

**Fig. 4:**
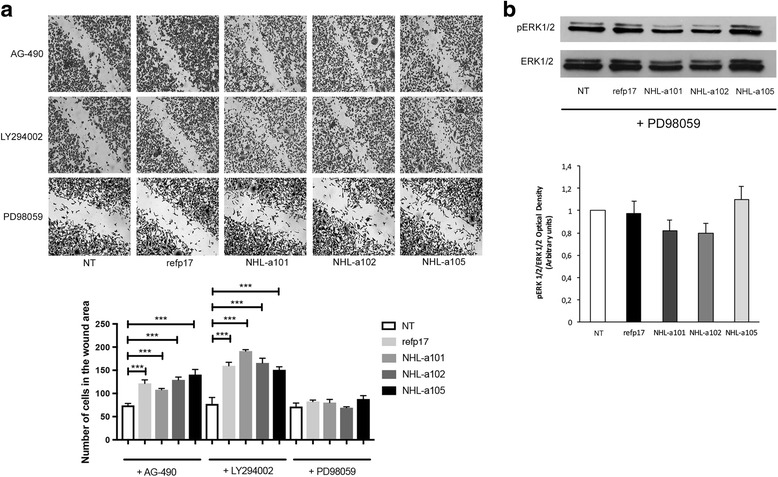
Role of MEK/ERK signaling pathway in migration induced by refp17 and vp17s. MDA-MB 231 cells were serum starved for 24 h in the presence or absence of the PI3K/Akt inhibitor LY294002 (20 μM), the Jak/STAT inhibitor AG-490 (20 μM), or the MEK/ERK1/2 inhibitor PD98059 (10 μM). **a** Confluent cell monolayers were serum starved for 24 h and then scratched with a 200 μl pipette tip. Cells were then incubated for 6 h in the absence (NT) or in the presence of 10 ng/ml of refp17 or vp17s. Images are representative of three independent experiments with similar results (original magnification 10×). Statistical analysis was performed by one-way ANOVA and the Bonferroni’s post-test was used to compare data (*** *p* < 0.001). **b** MDA-MB 231 cells were stimulated or not (NT) for 30 min with 200 ng/ml of refp17 or vp17s and then lysed. Equal amounts of total cellular extracts were analyzed for expression of pERK1/2 or ERK1/2 by western blot analysis using mAbs to pERK1/2 (Thr202, Tyr204) or ERK1/2 as specific reagents. Phosphorylation of ERK1/2 was verified by densiometric analysis and plotting of the pERK1/2/ERK1/2. Upper panel, Blots from one representative experiment of three with similar results are shown. Lower panel, Values reported for ERK1/2 are the mean ± SD of three independent experiments. Statistical analysis was performed by one-way ANOVA, and the Bonferroni post-test was used to compare data

To investigate further whether ERK1/2 pathway plays a role in refp17- and vp17s-induced migration activity, MDA-MB 231 cells were serum-starved for 24 h in the presence or absence of an optimal concentration of the inhibitors of PI3K/Akt (LY294002; 20 μM), Jak/STAT (AG-490; 20 μM) and MEK/ERK (PD98059; 10 μM). Then, confluent cell monolayers were scratched and incubated with the viral proteins for 6 h at 37 °C. As shown in Fig. 4a, cell migration in refp17- and vp17s-stimulated MDA-MB 231 cells was significantly inhibited by PD98059. At the same time, the viral proteins activity on MDA-MB 231 cell migration was not affected by LY294002 and AG-490. To confirm this evidence, we analyzed cells lysates of cells pretreated with PD98059 and then stimulated for 30 min with the viral proteins. As shown in Fig. 4b the activation of ERK1/2, previously observed upon viral proteins stimulation, was completely abolished in cells pretreated with the MEK/ERK inhibitor. Our results suggest that MEK/ERK1/2 pathway is required and critical for refp17 and vp17s-induced MDA-MB 231 cell migration.

### Vp17s, but not refp17, promote cancer anchorage-independent growth

**Fig. 5:**
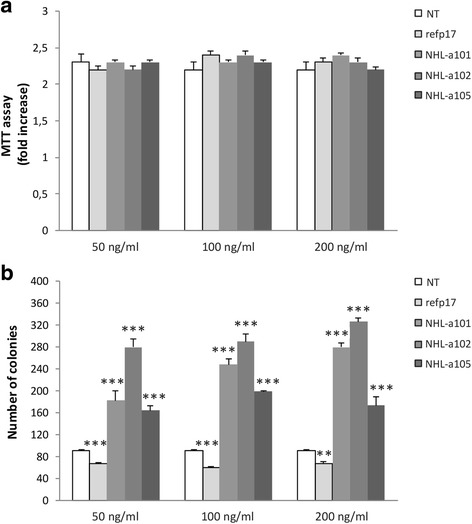
Effects of refp17 and vp17s on viability and colony-forming capacity of MDA-MB 231 cells. **a** MTT assay was used to determine the viability of MDA-MB 231 cells treated or not for 48 h with refp17 or vp17s as indicated. Data represent the average of three independent experiments performed in triplicate. Statistical analysis was performed by one-way ANOVA, and the Bonferroni post-test was used to compare data. **b** The effect of refp17 and vp17s on breast cancer cells clonogenicity was analyzed by soft agar assay. Cells were plated in six-well plate and, after two days, the medium was replaced using fresh medium with various concentration of refp17 or vp17s (range from 50 to 200 ng/ml). Not treated cells (NT) were used as a negative control. Data represent the average number of colonies ± SD from three independent experiments performed in triplicate. The statistical significance between control and treated cultures was calculated using two-way ANOVA, and the Bonferroni post-test was used to compare data (** *p* < 0.01, *** *p* < 0.001)

The effect of refp17 and vp17s on cell viability, was assessed by MTT assay. As shown in Fig. 5a, the treatment with the viral proteins for 48 h did not induced any toxic effect on MDA-MB 231 cells at any tested dose. The viral proteins were then investigated for their ability to enhance clonogenic activity of MDA-MB 231 cells. As shown in Fig. 5b, the vp17s NHL-a101, NHL-a102 and NHL-a105, at concentration ranging from 50 to 200 ng/ml, significantly increased the number of breast cancer cell colonies in soft agar, compared with NT cells. By contrast, refp17 significantly inhibited the colony-forming ability of breast cancer cells compared to NT cultures. These data indicate that the viral proteins treatment is not toxic for the MDA-MB 231 cells and, in agreement with previous results [36], underline the opposite effects of refp17 and vp17s in modulating MDA-MB 231 cell growth and clonogenicity.

## Discussion

The epidemiology of breast cancer in HIV^+^ women is rapidly changing. In the era of cART, the breast cancer incidence in HIV^+^ women approaches the general female population [11–14] but a younger median age is observed in HIV^+^ women compared to general population at the time of breast cancer diagnosis [17]. Furthermore**,** recent studies highlight a strong relationship between HIV-1 infection and stage of breast cancer at diagnosis: more HIV^+^ women show an advanced tumor stage than general population [17], and this occurs in the absence of any association with viral load or CD4^+^ T cell count [13, 14]. These findings show that different factors from immunodeficiency are likely candidates to contribute to breast cancer pathogenesis in HIV^+^ patients and point to the importance of further research into breast cancer in the HIV-1 setting. A key role of HIV-1 proteins is emerging in different pathologies, including cancer [43] so that it is likely to hypothesize their contribution to breast cancer aggressiveness and spreading.

Recent studies highlighted a key role of p17 in lymphoma development [44]. In particular, p17 expression in different tissues and organs and its known capability of promoting angiogenesis and lymphangiogenesis by activating an autophagy-based pathway [45] has been linked to processes of tumor growth and metastasis [43]. Moreover, vp17s endowed with a potent B-cell growth promoting and transforming activity have been detected in plasma and PBMCs of HIV^+^ patients with NHL [37, 46].

Data presented in this study show that both refp17 and some vp17s isolated from plasma of HIV^+^ patients with NHL strongly enhance MDA-MB 231 cell migration and invasiveness. The most aggressive breast cancer cell behavior was mediated by MAPK pathway activation following viral proteins interaction with CXCR2. In fact, the phosphorylation status of ERK1/2 increased in MDA-MB 231 cells treated with either refp17 or vp17s, whereas the specific ERK-dependent pathway inhibitor PD98059, targeting the upstream kinase MEK1, strongly impaired the p17-driven cell migratory activity. Our data are in agreement with previous studies showing that activation of the ERK1/2 pathway promotes cell motility [47] and invasiveness [48]. In addition, they show a quite similar biological activity between refp17 and vp17s with IL-8 in promoting MDA-MB 231 cell invasiveness. In fact, also IL-8 exerts its activity on the triple negative cancer cell line by activating the MEK/ERK signaling pathway following its interaction with CXCR1 and CXCR2 [49].

The one striking difference between refp17 and vp17s resides in the clonogenic activity exerted on MDA-MB 231 by vp17s only. In previous reports, a marked activation of the Akt signaling pathway was found to be promoted by vp17s − but not by refp17 − on B-cell lymphoma cells [36, 37]. In this study, both refp17 and vp17s showed a dramatic down-modulation of the Akt signaling pathway in MDA-MB 231 cells. At the same time, refp17 and vp17s did not show any STAT3 activation but all were effective in activating ERK1/2 compared to untreated cells. This suggests the presence of unidentified mechanisms at work for vp17s in promoting breast cancer cell clonogenicity. It is worth noting that all vp17s endowed with B-cell and breast cancer cell clonogenic activity are misfolded, compared to refp17 [37, 50]. Therefore, it is likely to hypothesize the presence of a specific epitope(s) involved in tumor cell clonogenic activity, which is exposed in vp17s and masked in refp17, and possibly binding to an alternate receptor(s). Further studies are needed to address this crucial question. Interestingly, although misfolded, vp17s were found to exert a refp17-like angiogenic and lymphangiogenic activity in vitro and in vivo [36, 45, 51]. Collectively, all this evidence corroborates the hypothesis that vp17s, because of their peculiar biological properties on both breast cancer cells and endothelial cells, are the most favorable microenvironmental proteins to promote breast cancer aggressiveness and spreading in HIV^+^ patients.

## Conclusion

Although limited to one single cell line here we provide the first evidence that refp17 and vp17s may play a key role in promoting human breast cancer cell migration and invasion, whereas vp17s may also affect breast cancer cell growth and transformation. Therefore, targeting p17 by specific neutralizing antibodies [52] or drugs [53] may be beneficial for treatment and better prognosis of breast cancer in the HIV-1 setting.

## Abbreviations

AIDS: Acquired immunodeficiency syndrome
Akt: Serine/threonine protein kinase
cART: Combined antiretroviral therapy
cs-FBS: Charcoal stripped-fetal bovine serum
CXCR1-2: C-X-C motif chemokine receptor 1-2
DMSO: Dimethyl sulfoxide
EGTA: Ethylene glycol-bis(β-aminoethyl ether)-N,N,N′,N′-tetraacetic acid
ER: Estrogen receptor
ERK: Extracellular signal-regulated kinase
FBS: Fetal bovine serum
Gag: Group-specific antigen
HEPES: 4-(2-hydroxyethyl)-1-piperazineethanesulfonic acid
HER2: Human epidermal growth factor receptor
HIV-1: Human immune deficiency virus type 1
IL-8: Interleukin-8
Jak: Janus kinase
mAb: Monoclonal antibody
MAPK: Mitogen-activated protein kinases
MTT: 3-(4,5-dimethylthiazol-2-yl)-2,5-diphenyltetrazolium
PI3K: Phosphatidylinositol-4,5-bisphosphate 3-kinase
PR: Progesterone receptor
STAT: Signal transducer and activator of transcription
